# Supplementary material for: Towards effective clinical decision support systems: A systematic review
Source: PLoS One. 2022 Aug 15;17(8):e0272846. doi: 10.1371/journal.pone.0272846 (PMC9377614; doi:10.1371/journal.pone.0272846)
Supplement: S1 File — (DOCX) [file pone.0272846.s001.docx]

| Study | Year | Data Source | Country | Setting | Study Design | Recipient of intervention | Purpose of care | Knowledge Management | Technological Features | System integration | Type of System | Simon’s phase |
| --- | --- | --- | --- | --- | --- | --- | --- | --- | --- | --- | --- | --- |
| Catalani 2014 | 2014 | PlosOne | Kenya | Hospital Academic Center | Prototype development; effectiveness | Physicians; patient | Patient-specific treatment: HIV and tuberculosis care | Clinical practice guidelines; rule-based module; algorithm | Error reduction; cost and time reduction | Electronic Health Record system | Specific computerized tool | Implementation |
| Jiang 2019 | 2019 | PlosOne | United States of America | Any healthcare setting | Cross-sectional | Patient | Patient-specific diagnosis: breast cancer metastasis | Bayesian networks; Algorithm; if/then statements; variables-based | Assessment; Recommendation and suggestion | Electronic Health Record system | Specific computerized tool | Choice |
| Akhloufi 2019 | 2019 | PlosOne | Netherland | Hospital Academic Center | Development; effectiveness | Physicians; patient | Medication/drug prescription | Clinical practice guidelines; rule-based module | Recommendation and suggestion | Electronic Health Record system; Computerized Provider Order Entry system | Web-based application | Design |
| Dalaba 2014 | 2014 | PlosOne | Ghana | Any healthcare setting | Cross-sectional; Case Study; Cost analysis; Implementation | Nurses; patient | Specific workflow: antenatal and delivery care | Algorithmic logic; clinical practice guidelines | Recommendation and suggestion; alerts, notifications and reminders | Standalone CDSS | Software application | Intelligence |
| Sim 2017 | 2017 | PlosOne | Singapore | Outpatient setting | Pilot study | physicians | Patient-specific disease: diabetes care | Algorithmic logic; clinical practice guidelines | Alerts, notifications and reminders; Information management and monitoring; Error reduction | Electronic Health Record system | Web-based application; data analytics | Intelligence |
| Berrouiguet 2016 | 2016 | PlosOne | Spain | Community pharmacy | Observational study; development | Healthcare provider | Medication/drug prescription | Variables-based | Assessment; Information management and monitoring | Electronic Health Record system | Web-based application | Design |
| Tamburrano 2020 | 2020 | PlosOne | Italy | Hospital Academic Center | Observational study; cost-effectiveness | physicians | Laboratory tests | Rule-based module | Cost and time reduction; standardization; alerts, notifications and reminders | Computerized Provider Order Entry system | Specific computerized tool | Intelligence |
| Breitbart 2020 | 2020 | PlosOne | Germany | Clinical network | Feasibility study; effectiveness | General practitioners | Patient-specific diagnosis: skin disease | Not identified | Standardization; error reduction | Standalone CDSS | Web and mobile application | Intelligence |
| De Lima Marinho 2019 | 2019 | PlosOne | Finland | Hospital Academic Center |  | patient | Patient-specific diagnosis: respiratory disorders | Not identified | Information management and monitoring | Standalone CDSS | Machine learning-based | Intelligence |
| Bernasconi 2019 | 2019 | PlosOne | Nigeria | Any healthcare setting | Cross-sectional; implementation; effectiveness | General practitioners | Specific workflow: Boko Haram insurgency | Algorithmic logic; clinical practice guidelines | Information management and monitoring | Standalone CDSS | Web and mobile application | Implementation |
| Menon 2020 | 2020 | Nature | United States of America | Hospital | Pilot study | General practitioners | Patient-specific diagnosis: pediatric acute kidney injury | Clinical practice guidelines | Assessment; Information management and monitoring; alerts, notifications and reminders | Electronic Health Record system | Specific computerized tool | Intelligence |
| Kim 2020 | 2020 | Nature | Korea | Any healthcare setting | Assessment | General practitioners | Specific workflow: clinic-genomic | Rule-based module; knowledge base; algorithmic logic | Assessment | Standalone CDSS | Data-layer infrastructure | Intelligence |
| Falciglia 2020 | 2020 | Nature | United States of America | Hospital | Effectiveness | General practitioners | Specific workflow: Nutrition | Not identified | Calculation and scoring; error reduction | Electronic Health Record system | Software application | Intelligence |
| Arain 2020 | 2020 | Nature | United States of America | Hospital | Assessment | General practitioners | Specific workflow: phototherapy initiation in preterm infants | Clinical practice guidelines | Recommendation and suggestion; process automation and prioritization | Electronic Health Record system | Web-based application | Intelligence |
| Seroussi 2013 | 2013 | Nature | France | Hospital | Assessment | physicians | Patient-specific treatment: breast cancer | Clinical practice guidelines | Recommendation and suggestion; alerts, notifications and reminders | Standalone CDSS | Web-based application | Intelligence |
| Piri 2017 | 2017 | DSS | United States of America | Hospital | Development | General practitioners | Patient-specific disease: diabetes retinopathy | Variable-based; neural artificial network | Recommendation and suggestion; prediction | Electronic Medical Record system | Data Analytics | Design |
| Lin 2006 | 2006 | DSS | United States of America | any healthcare setting | Design; Implementation; effectiveness | Clinicians | Patient-specific diagnosis: lower back pain | Knowledge base; rule-based module; inference engine | Assessment; Recommendation and suggestion | Standalone CDSS | Web-based application | Design |
| Rao 2000 | 2000 | DSS | United States of America | any healthcare setting | Proposal | Physicians | General | Inference engine; algorithmic logic; rule-based module | Standardization | Specific information system | Specific computerized tool | Design |
| Valkenhoef 2013 | 2013 | DSS | Netherland | Community pharmacy | Development | physicians | Clinical trial | Standardized Clinical Terminologies; rule-based module | Process automation and prioritization; information management and monitoring | Specific information system | Software application | Design |
| Akcura 2014 | 2014 | DSS | United States of America | Any healthcare setting | Proposal | Physicians | Medication/drug prescription | Bayesian network; clinical practice guidelines | Events; error reduction; information management and monitoring | Computerized Provider Order Entry system | Specific computerized tool | Design |
| Hu 2006 | 2006 | DSS | United States of America | Any healthcare setting | Experimental; effectiveness | General practitioners | Specific workflow: Patient image pre-fetching | Knowledge base; rule-based module; variables-based | Information management and monitoring | Specific information system | Image Retrieval Expert System | Intelligence |
| Piri 2020 | 2020 | DSS | United States of America | Any healthcare setting | Experimental study | General practitioners; patient | Patient-specific disease: Parkinson | Variables-based; standardized clinical terminology; neural networks | Recommendation and suggestion; error reduction; prediction | Electronic Health Record system | Specific computerized tool | Intelligence |
| Michalowski 2003 | 2003 | DSS | Canada | Emergency department | Design; prototype development | Healthcare provider | Triage | Algorithmic logic; rule-based module; data mining techniques | Recommendation and suggestion | Standalone CDSS | Mobile application | Design |
| Johnson 2014 | 2014 | DSS | United States of America | Any healthcare setting | Technology acceptance | Healthcare provider | Patient-specific disease: chronic diseases | Algorithmic logic; clinical practice guidelines | Alerts, notifications and reminders | Electronic Health Record system | Web-based application | Choice |
| Yao 2013 | 2013 | DSS | United States of America | Any healthcare setting | Proposal; Proof of concept prototype | General practitioners | Clinical pathways | Clinical practice guidelines; rule-based module; knowledge base; inference engine | Recommendation and suggestion; alerts, notifications and reminders | Specific information system | Web-based application | Design |
| Lussier 2007 | 2007 | DSS | United States of America | Any healthcare setting | Design; prototype development | General practitioners | general | Rule-based module; standardized clinical terminology; knowledge base | Alerts, notifications and reminders; events | Standalone CDSS | Mobile application | Design |
| Corny 2020 | 2020 | PubMed | France | Hospital | Development; effectiveness | Clinical pharmacist | Medication/drug prescription | rule-based module; algorithmic logic | Error reduction; process automation and pioritization; alerts, notifications and reminders | Electronic Health Record system; Computerized Provider Order Entry system | Machine learning-based | Choice |
| Altay 2020 | 2020 | PubMed | Turkey | Hospital | Confirmatory; cost-effectiveness | Physicians; patient | Patient-specific disease: liver fibrosis | Algorithmic logic; rule-based module; data mining techniques | Prediction | Specific information system | Artificial intelligence-based | Choice |
| Cheung 2020 | 2020 | PubMed | Canada | Hospital Academic Center | Effectiveness | Clinicians; patient | Patient-specific treatment: asthma | Clinical practice guidelines | Prediction; assessment; error reduction; information management and monitoring; alerts, notifications and reminders; recommendation and suggestion | Electronic Medical Record system | Web-based application | Choice |
| Yoo 2020 | 2020 | PubMed | Korea | Emergency department | Descriptive study; assessment | Physicians; patient | Medication/drug prescription | Rule-based module; knowledge base; algorithmic logic | Alerts, notifications and reminders; events | Computerized Provider Order Entry system | Web and mobile application | Design |
| Downie 2020 | 2020 | PubMed | Australia | Community pharmacy | Feasibility study; prototype development | Clinical pharmacist | Patient-specific diagnosis: low back pain | Knowledge base; inference engine; clinical practice guidelines | Recommendation and suggestion; error reduction | Standalone CDSS | Web-based application | Design |
| Lee 2020 | 2020 | PubMed | Korea | Hospital | Descriptive study; effectiveness | Healthcare provider; patient | Patient-specific treatment: cancer | Not identified | Recommendation and suggestion | Standalone CDSS | Artificial intelligence-based | Intelligence |
| Hamedan 2020 | 2020 | PubMed | Iran | Hospital Academic Center | Development; effectiveness | nephrologists | Patient-specific disease: chronic diseases | rule-based module; if/then statements; variable-based | Prediction; information management and monitoring | Standalone CDSS | Software application | Choice |
| Ucuz 2020 | 2020 | PubMed | Turkey | Outpatient setting | Effectiveness | psychiatrists | Specific workflow: juvenile delinquency | Standardized Clinical Terminologies | Prediction; information management and monitoring | Standalone CDSS | Artificial intelligence-based | Intelligence |
| Wu 2020 | 2020 | PubMed | China | Hospital | Effectiveness | General practitioners | Specific workflow: Covid-19 triage | algorithmic logic | Prediction; calculation and scoring; assessment | Standalone CDSS | Machine learning-based | Intelligence |
| Choi 2020 | 2020 | PubMed | Korea | Hospital | Development; effectiveness | patient | Patient-specific treatment: hepatocellular carcinoma | Variable-based; algorithmic logic | Recommendation and suggestion; prediction | Standalone CDSS | Machine learning-based | Choice |
| Sepúlveda 2020 | 2020 | PubMed | China | Clinical network | Cross-sectional; effectiveness | Oncologists | Patient-specific treatment: breast cancer | Clinical practice guidelines; variable-based; inference engine; knowledge base | Recommendation and suggestion; calculation and scoring | Standalone CDSS | Artificial intelligence-based | Choice |
| Schaaf 2020 | 2020 | PubMed | Germany | Hospital Academic Center | Qualitative study; development | General practitioners | Specific workflow: rare diseases | Knowledge base | Recommendation and suggestion | Standalone CDSS | Software application | Design |
| Xu 2020 | 2020 | PubMed | China | Community pharmacy | Proposal | Oncologists | Specific workflow: oncology pharmacotherapy | Knowledge base | Events; process automation and prioritization | Standalone CDSS | Web-based application | Design |
| Holmstrom 2020 | 2020 | PubMed | Sweden | Emergency Department | Qualitative study | nurses | Triage | rule-based module | Recommendation and suggestion; process automation and prioritization; assessment; | Standalone CDSS | Specific computerized tool | Intelligence |
| Chin 2020 | 2020 | PubMed | United States of America | Hospital Academic Center | Development; effectiveness | physicians | Medication/drug prescription | Rule-based module; knowledge base; algorithmic logic | Alerts, notifications and reminders; Information management and monitoring; Error reduction | Electronic Health Record system | Software application | Design |
| Lichtenegger 2020 | 2020 | PubMed | Austria | Inpatient setting | Effectiveness | Nurses; physicians | Patient-specific disease: diabetes care | Algorithmic logic | Process automation and prioritization; standardization; Recommendation and suggestion; calculation and scoring | Standalone CDSS | Mobile application | Choice |
| Niazkhani 2020 | 2020 | PubMed | Iran | Outpatient setting | development | Nephrologists | Specific workflow: kidney transplant care | Rule-based module; if/then statements; knowledge base; algorithmic logic | Error reduction; alert, notifications and reminders; Recommendation and suggestion | Computerized Provider Order Entry system | User interface | Design |
| Torres Silva 2020 | 2020 | PubMed | Colombia/United States of America | Clinical network | Development | General practitioners | Specific workflow: antenatal care | Clinical practice guidelines; inference engine | Recommendation and suggestion; process automation and prioritization; Information management and monitoring; alerts, notifications and reminders | Standalone CDSS | Web and mobile application | Design |
| Vargason 2016 | 2016 | AIS e-library | United States of America | Any healthcare setting | Development; experimental | Clinicians | Patient-specific disease: atypical pleural effusion | Algorithmic logic | Information management and monitoring | Standalone CDSS | Software application | Design |
| Berge 2017 | 2017 | AIS e-library | Norway | Hospital | Prototype development | physicians | Specific workflow: anaesthesia allergies during surgery | Algorithmic logic; rule-based module; data mining techniques | Information management and monitoring | Electronic Health Record system | Machine learning-based | Design |
| Miller 2019 | 2019 | AIS e-library | United States of America | Emergency department | Cross-sectional; development | Clinicians; patient | Specific workflow: adolescent sexual health care | Clinical practice guidelines | Recommendation and suggestion | Standalone CDSS | Software application | Design |
| Xu 2018 | 2018 | AIS e-library | United States of America | Any healthcare setting | Prototype development; implementation | General practitioners | Specific workflow: genetic | Algorithmic logic | Information management and monitoring | Standalone CDSS | Cloud computing | Design |
| Wasylewicz 2018 | 2018 | AIS e-library | Australia | Any healthcare setting | Case study; research in progress | General practitioners | general | Clinical practice guidelines; rule-based module; knowledge base; inference engine | Recommendation and suggestion; information management and monitoring | Standalone CDSS | Data analytics | Design |
| Bond 2017 | 2017 | AIS e-library | Australia | Hospital | Effectiveness | General practitioners | Specific workflow: antimicrobial stewardship | Clinical practice guidelines | Recommendation and suggestion | Standalone CDSS | knowledge-based | Intelligence |
| Strockbine 2020 | 2020 | AIS e-library | United States of America | Hospital Academic Center | effectiveness | General practitioners | Specific workflow: phlebotomy testing | Clinical practice guidelines | Information management and monitoring | Electronic Health Record system; Computerized Provider Order Entry system | Specific computerized tool | Intelligence |
| Smit 2017 | 2017 | AIS e-library | Netherland | Hospital Academic Center | Exploratory | physicians | general | Clinical practice guidelines; rule-based module | Recommendation and suggestion | Standalone CDSS | Knowledge-based | Intelligence |
