# Supplementary material for: Towards effective clinical decision support systems: A systematic review
Source: PLoS One. 2022 Aug 15;17(8):e0272846. doi: 10.1371/journal.pone.0272846 (PMC9377614; doi:10.1371/journal.pone.0272846)
Supplement: S3 File — (DOC) [file pone.0272846.s003.doc]

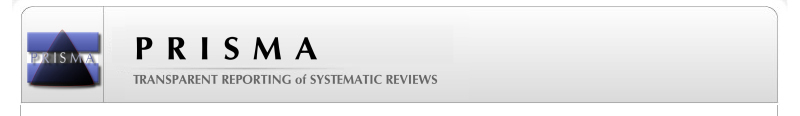
**PRISMA 2009 Flow Diagram**

**Screening**

**Included**

**Eligibility**

**Identification**

PubMed = 322

DSS = 245

Nature = 115

AIS e-library = 52

Plos One = 34

(n = 67)

Records
(n = 768)

Records screened
(n = 690)

Records excluded (n = 598):

No open access

No articles

Out of period

No English

Full-text articles assessed for eligibility
(n = 92)

Full-text articles excluded
(n = 40):

No CDSS

Not specific CDSS

Literature Review articles

Studies included in synthesis

(n = 52)

Duplicates recorded
(n = 78)
