# Supplementary material for: Towards effective clinical decision support systems: A systematic review
Source: PLoS One. 2022 Aug 15;17(8):e0272846. doi: 10.1371/journal.pone.0272846 (PMC9377614; doi:10.1371/journal.pone.0272846)
Supplement: Attachment — Submitted filename: Response to Reviewers.docx [file pone.0272846.s004.docx]

**Response to Reviewers**

**Reviewer #1:**

**A:** First of all, we would like to thank for the comments and constructive suggestions that will certainly contribute to the enrichment of this study. In this revised version, we reinforce all the points suggested by the reviewer, especially in the methods and in the discussion part. The changes are highlighted in yellow in the revised manuscript with track changes version.

In the 'Information Sources' chapter, we reinforce the criteria that were applied to choose data sources, based on the Scimago ranking. The search strategy was also completed, reinforcing the filters and terms used for the search. We also combine the search strategy with the eligibility criteria.

In the discussion part, we refer to other systematic review studies that address the Clinical Decision Support System (DSS), but that do not relate to the objective of this study. They tend to analyse the application/performance of DSS into specific clinical problems, while we intend to analyse and characterize DSS by their capacities supporting the phases decision making process.

In the Discussion and Conclusion chapters, we rewrote as suggested, presenting a clearer, literature-based structure. We hope that the changes made are as expected.

**Reviewer #2:**

**A:** First of all, we would like to thank for the comments and constructive suggestions that will certainly contribute to the enrichment of this study. In this revised version, we reinforce all the points suggested by the reviewer, especially in the methods part. The changes are highlighted in green in the revised manuscript with track changes version.

In the Data Extraction and Management chapter, we describe all the information that was extracted from the reviewed articles. In addition, we highlighted what was expected to be found at each stage of the decision-making process. We included the list of conditions considered for classifying DSS according to these phases (Table 2). We hope that the changes made are as expected.
